# Reducing Neuroinflammation via Inhibition of Fibrinogen Deposition and Microglial Activation as an Underlying Mechanism of Paning I Decoction in Ameliorating Parkinson's Disease Symptoms

**DOI:** 10.1002/iid3.70310

**Published:** 2026-02-05

**Authors:** Yan‐Jun Chen, Jing‐Wen Chen, Ming‐Rong Xie, Rui‐Zhen Wang, Yu‐Ling Wan, Jie Zeng, Bing‐Wu Zhong, Sheng‐Qiang Zhou, Fang Liu

**Affiliations:** ^1^ Hunan University of Chinese Medicine Changsha China; ^2^ National TCM Master Liu Zuyi Inheritance Studio Hunan Provincial Hospital of Integrated Traditional Chinese and Western Medicine Changsha China; ^3^ Nanjing University of Chinese Medicine Nanjing China; ^4^ The Second Xiangya Hospital Central South University Changsha China

**Keywords:** fibrinogen, microglia, neuroinflammation, Paning I decoction, Parkinson's disease

## Abstract

**Background:**

Parkinson's disease (PD) is a major neurodegenerative disorder. Some patients show limited response to standard therapies, driving the need for new complementary treatments. Paning I decoction (PNID), a traditional herbal formula, has shown the potential to alleviate PD symptoms, but its exact mechanisms remain unclear.

**Methods:**

We tested PNID in a mouse model of PD induced by a 1‐methyl‐4‐phenyl‐1,2,3,6‐tetrahydropyridine (MPTP). Mice received different PNID doses to evaluate symptom alleviation. We used behavioral tests and laboratory analyses to study brain changes, including neuron damage assessment, dopamine and tyrosine hydroxylase (TH) measurements, blood–brain barrier (BBB) integrity and microglia evaluation, and inflammatory marker analysis.

**Results:**

PNID treatment alleviated PD symptoms in a dose‐dependent manner. High‐dose PNID performed similarly to Madopar. Neuron protection: increased dopamine and TH expression. BBB repair: less leakage and fibrinogen accumulation. Regulation of polarization: shifted microglia from M1 to M2 states. Inflammation control: lowered pro‐inflammatory factors (IL‐6, IL‐1β, and TNF‐α) while increasing anti‐inflammatory factors (IFN‐β, IL‐10, and IL‐4).

**Conclusions:**

PNID may serve as a promising complementary therapy for PD. Benefits may come from the repair of the BBB, reduction of fibrinogen deposition, and decline in neuroinflammation by modulating microglial polarization.

## Graphical abstract

Paning I decoction alleviated neuroinflammation in Parkinson's disease.

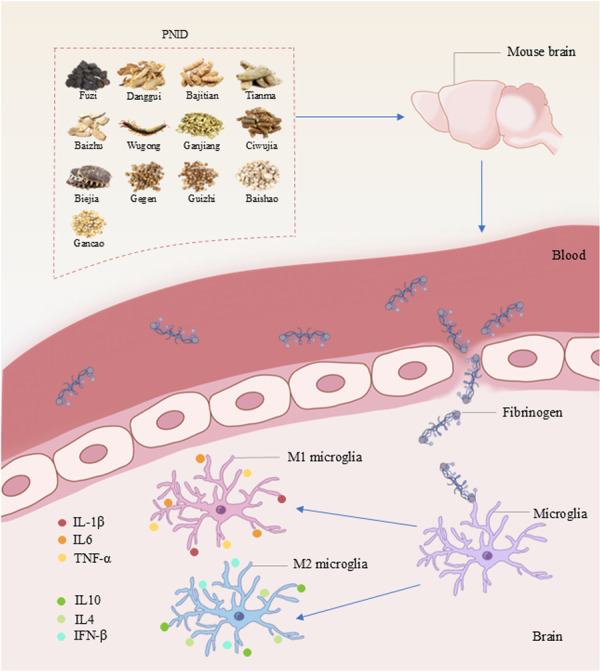

**Abbreviations**
Arg‐1: arginase 1
BBB: blood–brain barrier
CNS: central nervous system
DA: dopamine
DOPAC: dihydroxyphenylacetic acid
HPLC: high performance liquid chromatography
HVA: homovanillic acid
IBA‐1: ionized calcium binding adapter molecule 1
IFN‐β: interferon‐β
IL‐10: interleukin‐10
IL‐1β: interleukin‐1β
IL‐4: interleukin‐4
IL‐6: interleukin‐6
MHC‐II: major histocompability antigen‐II
MPTP: 1‐methyl‐4‐phenyl‐1,2,3,6‐tetrahydropyridine
OX‐42: clone name of monoclonal antibody of complement receptor type
PD: Parkinson's disease
PNID: Paning I decoction
SNpc: substantia nigra pars compacta
TCM: traditional Chinese medicine
TH: tyrosine hydroxylase
TNF‐α: tumor necrosis factor α

## 1 Introduction

Parkinson's disease (PD) represents a prevalent neurodegenerative condition distinguished by the progressive loss of dopaminergic neurons within the substantia nigra pars compacta (SNpc) and a consequent decline in dopamine (DA) concentrations in the striatum [1]. The detailed pathogenic mechanism of PD is largely undefined. Neuroinflammation is considered one of its possible pathogenic factors [2, 3], due to its essential role in neuronal loss and DA reduction [4]. Therapeutic strategies targeting neuroinflammation, such as PD01A [5] and nonsteroidal anti‐inflammatory drugs [6], showed promising efficacy. Recently, interest has grown in exploring the treatment of PD derived from traditional Chinese medicine (TCM). Several TCM formulations demonstrated beneficial effects in managing PD symptoms associated with various TCM syndromes, including Pingchan granule [7] and Da Dingfengzhu [8]. Some patients show limited response to standard PD therapies, underscoring the pressing need to investigate and promote additional effective complementary and alternative therapeutic agents.

Paning I decoction (PNID) is a traditional formula developed by Liu Zuyi, a famous modern master of TCM, based on Sini decoction and Guizhijiegegen decoction. PNID is constructed based on the TCM principle of tonifying Yang qi and promoting blood circulation. When used as a complementary medication in PD patients, PNID showed promising therapeutic effects [9]. PNID consists of 13 natural medicines, including Fuzi (*Aconitum carmichaelii Debeaux*), Danggui (*Angelica sinensis*), Bajitian (*Gynochthodes officinalis*), Tianma (*Gastrodia elata Blume*), Baizhu (*Atractylodes macrocephala*), Wugong (*Scolopendridae*), Ganjiang (*Zingiber officinale Roscoe*), Ciwujia (*Eleutherococcus senticosus*), Biejia (*Trionycis Carapax*), Gegen (*Pueraria montana var. lobata*), Baishao (*Paeonia lactiflora Pall*.), Guizhi (*Neolitsea cassia*), and Gancao (*Glycyrrhiza glabra L*.). Previous studies exhibited that most of the chemical components found in these natural medicines have anti‐inflammatory properties, such as higenamine [10], monotropein [11], atractylenolide‐1 [12], gastrodin [13], and liquiritin [14], indicating the potential role of PNID in inhibiting neuroinflammation and alleviating symptoms associated with PD.

Considering the potential of PNID in suppressing inflammation and relieving PD symptoms, we observed how fibrinogen and microglia are involved in the neuroinflammatory response and further validated the therapeutic efficacy of PNID. Fibrinogen is barely detectable in normal brain tissue. Unlike previous studies on the involvement of microglia in neuroinflammation, we observed that the blood–brain barrier (BBB) was damaged and fibrinogen leakage into the central nervous system (CNS) activated microglia to participate in the inflammatory response.

## 2 Materials and Methods

We established a chronic PD model in C67BL6 mice by intraperitoneal injection of 1‐methyl‐4‐phenyl‐1,2,3,6‐tetrahydropyridine (MPTP), followed by PNID treatment. At the end of the experiment, neurons, DA, TH, BBB damage, fibrinogen, microglia, and inflammatory factors were detected.

### 2.1 Preparation of PNID

The ingredients for the preparation of PNID included 9 g Fuzi, (Sichuan, China, Batch Number 22032404), 15 g of Bajitian (Guangdong, China, Batch Number 220101), 15 g of Ciwujia (Hunan, China, Batch Number 22111403), 15 g of Tianma (Yunnan, China, Batch Number 2021100702), 15 g of Biejia (Hunan, China, Batch Number 220601), 9 g of Ganjiang (Fujian, China, Batch Number 23052001), 15 g of Guizhi (Guangxi, China, Batch Number 22121201), 15 g of Baishao (Anhui, China, Batch Number 211201), 15 g of Baizhu (Zhejiang, China, Batch Number 2022121904), 15 g of Gegen (Anhui, China, Batch Number 210701), 9 grams of Danggui (Gansu, China, Batch Number 22111701), 5 g of Wugong (Hubei, China, Batch Number 220801), and 9 g of Gancao (Neimenggu, China, Batch Number 220901). All herbs were identified by pharmacist Qixue Tian from Hunan Provincial Hospital of Integrated Traditional Chinese and Western Medicine, ensuring their authenticity and quality. Fuzi was first boiled for 1 h, then the remaining drug was added and decocted for 30 min to obtain the aqueous extract of PNID.

Ultra‐performance liquid chromatography combined with high‐resolution mass spectrometry (UHPLC‐HRMS) was used. The chromatographic conditions were as follows: the chromatographic column was ACQUITY UPLC HSS T3 (2.1 mm × 100 mm, 1.8 µm). 0.1% formic acid aqueous solution and 0.1% formic acid acetonitrile solution were used as mobile phases. The flow rate was 0.3 mL/min and the column temperature was 35°C. Thermo Q‐Exactive HFX mass spectrometer was used for positive and negative ion electric ion acquisition mode (m/z ranges from 90 to 1300). The details of MS were: spray voltage: 3800, −3000; shealth gas: 45; aux gas: 20; spare gas: 0; capillary temperature: 320°C; probe heater temp: 370°C.

### 2.2 Experimental Animals

Male C57BL/6 mice, weighing ~20 ± 2 g, were obtained from Hunan Slake Jingda Laboratory Animal Company. These animals were housed in laboratory cages maintained at a temperature range of 18°C–25°C and relative humidity of 40%–70%, under conditions that allowed them unrestricted access to both food and water. The Experimental Animal Ethics Committee of Hunan University of Chinese Medicine has approved the conduct of this experiment (Ethical Approval Number: LL2022060101).

### 2.3 PD Model, Groups, and Interventions

To establish a mouse model of PD, MPTP (30 mg/kg) was injected intraperitoneally at a 3‐day interval, with each mouse receiving a total of 10 injections. A total of 90 mice were used in the experiments. Fifteen mice were randomly assigned as the control group and intraperitoneally injected with a normal saline solution. The remaining 75 mice were injected intraperitoneally with MPTP according to the injection plan and were randomly divided into 5 groups, with 15 mice in each group: (i) the model group; (ii) the Madopar group; (iii) the PNID low‐dose group (PNID‐L); (iv) the PNID medium‐dose group (PNID‐M); and (v) the PNID high‐dose group (PNID‐H). The treatment began on the day of the sixth injection of MPTP and continued for 14 consecutive days. The control group and the model group were given intragastric administration of normal saline. The Madopar group was given medobal solution by gavage (39 mg/kg). The PNID‐L, PNID‐M, and PNID‐H groups were administered intragastrically with PNID solutions at doses of 10.47, 20.93, and 41.86 g/kg, respectively. The dosage regimen for PNID was based on the body surface area ratio between humans and mice. For an adult weighing 70 kg, the daily clinical dosage of PNID medication is 161 g. For the PNID‐M group, the medium dose was set at 20.93 g/kg, which corresponded to the clinical dose. The dose in the PNID‐L group was 0.5 times of the median dose (10.47 g/kg), and the dose in the PNID‐H group was two times of the median dose (41.86 g/kg). Behavioral tests were conducted on the day of the final treatment administration (Figure 2A). One mouse in the PNID‐L group died during the treatment period. After the treatment period for the mice was completed, samples were collected the next day.

### 2.4 Behavioral Tests

#### 2.4.1 Pole Climbing Test

The pole test serves as an instrumental assessment to quantify balance adjustment proficiency and limb agility in murine models. Within the context of this investigation, mice were positioned with their cephalic regions oriented downwards atop a polished wooden pole, dimensioned at 0.1 m in diameter and 0.6 m in length. The temporal duration required for the mice to traverse the pole from the apex to the base was meticulously recorded.

#### 2.4.2 Gait Analysis

Assessment of locomotor function and gait patterns in mice frequently involves gait analysis. Here, mice were marked with black ink on their forepaws and red ink on their hind paws, and subsequently placed on a narrow runway measuring 10 cm in length and 2 cm in width. A pristine sheet of paper was positioned beneath the runway, and the mice were permitted to traverse the path in a single direction. Post‐traversal, the mice footprints were meticulously analyzed. Specifically, the distance separating consecutive footprints was designated as the step length, whereas the perpendicular distance between contralateral footprints represented the step width.

#### 2.4.3 Open‐Field Experiment

This test is a widely employed paradigm to observe spontaneous activity and movement patterns in mice. In this study, mice were introduced to the center of an open‐field arena comprising a grid of 9 squares, each dimensioned at 50 cm × 50 cm × 50 cm. The overall distance traversed, the count of squares crossed, and the locomotor trajectory of the mice were systematically documented using a specialized mouse open‐field video analysis system (Borealis, Hong Kong). The duration of observation was standardized at 4 min.

### 2.5 Transmission Electron Microscopy

Brain tissues were immobilized within an electron microscopy‐compatible fixative solution (Servicebio, G1102) to preserve cellular ultrastructure. After thorough rinsing, these tissues underwent a series of dehydration steps, followed by embedding in an appropriate medium. Ultrathin sections were then meticulously prepared using a precision microtome (Leica, Leica UC7). The resultant sections were subjected to a dual staining protocol involving lead and uranium compounds to enhance contrast under microscopic examination. Finally, these stained sections were analyzed using a high‐resolution transmission electron microscope (HITACHI, HT7700) to elucidate the ultrastructural features of the brain tissues.

### 2.6 Nissl Staining

Sections of midbrain SNpc in mice were subjected to xylene treatment for 10 min to facilitate dehydration, followed by immersion in ethanol for an additional 5 min to ensure thorough permeabilization. Subsequently, the tissues underwent rinsing with distilled water to remove any residual solvents. For histological staining, the tissues were incubated with Nissl stain (AWI0501, Wellbio). Poststaining, the tissues were mounted using buffer glycerol. The prepared slides were then examined under a high‐resolution light microscope (BA210T, Motic), enabling detailed observation and analysis of the neuronal morphology within the SNpc region.

### 2.7 High Performance Liquid Chromatography (HPLC)

DA, dihydroxyphenylacetic acid (DOPAC), and homovanillic acid (HVA) were analyzed by HPLC. Striatal tissue was weighed, homogenized by sonication in ice‐cold 0.01 mM perchloric acid containing 0.01% EDTA, then centrifuged and filtered. Separation employed a mobile phase (1.2 mL/min flow rate) consisting of: 85 mM citric acid, 100 mM anhydrous sodium acetate, 0.2 mM EDTA‐2Na, and 15% (v/v) methanol (pH 3.68). DA, DOPAC, and HVA concentrations (ng/mg tissue weight) were quantified via chromatogram analysis using standard calibration curves or the internal standard method.

### 2.8 Immunohistochemical Staining

Midbrain SNpc sections of mice were deparaffinized, rinsed in distilled water, and rinsed in PBS (AWI0137a, Wellbio) after heat‐induced antigen retrieval. Samples were incubated with anti‐TH (25859‐1‐AP, America), anti‐IL‐1β (ab9722, England), anti‐IL‐6 (bs‐0379R, China), anti‐TNF‐α (60291‐1‐Ig, America), anti‐IL‐4 (bs‐0581R, China), anti‐IL‐10 (bs‐0698R, China), and anti‐IFN‐β (bs‐23733R, China), rinsed with PBS (AWI0137a, Wellbio), and incubated with secondary antibodies (AWS0002, Abiowell). Color development was achieved by adding DAB (ZLI‐9017, ZSGB‐Bio), and counterstaining (AWI0009a, Wellbio) was performed using hematoxylin. Samples were observed under a microscope (BA410T, Motic).

### 2.9 Immunofluorescence

Midbrain SNpc sections of mice were deparaffinized, rinsed in distilled water, and rinsed in PBS (AWI0137a, Wellbio) after heat‐induced antigen retrieval. Samples were incubated with anti‐fibrinogen (ab189490, England), anti‐occludin (27260‐1‐AP, America), and anti‐clandin‐5 (35‐2500, China), rinsed with PBS (AWI0137a, Wellbio), and incubated with fluorescent secondary antibodies (AWS0002, Abiowell). DAPI (AWC0292a, Wellbio) was used to stain nuclei. Samples were observed by fluorescence microscopy (BA410T, Motic).

### 2.10 Western Blot Analysis

Midbrain SNpc of mice were lysed on ice and centrifuged. Proteins were separated and blotted to membranes, which were incubated with anti‐TH (25859‐1‐AP, Proteintech), anti‐IBA1 (10904‐1‐AP, Proteintech), anti‐OX42 (ab184308, Abcam), anti‐MHC‐II (Bs‐8481R, Bioss), anti‐Arg1 (16001‐1‐AP, Proteintech), and anti‐β‐actin (66009‐1‐Ig, Proteintech), rinsed with PBS (AWI0137a, Wellbio), and incubated with secondary antibodies (AWS0002, Abiowell). ECL was used for color exposure, and the images were analyzed using a gel imaging system (ChemiScope6100, China).

### 2.11 Statistical Analysis

Statistical analysis was performed using GraphPad Prism 9.0. Data are presented as mean ± standard deviation (SD). All data sets met the assumptions of normality and equal variances. For comparisons among multiple groups, a one‐way analysis of variance (ANOVA) is conducted, followed by Tukey's honestly significant difference post hoc test to control the family‐wise error rate (FWER) for all pairwise comparisons [15]. Tukey's method is selected to control the FWER while maintaining statistical power in the context of multiple comparisons. Post hoc test: Tukey's test is optimal for all pairwise comparisons in balanced designs, providing stricter control of type I error compared to uncorrected tests. Multiple comparisons correction: This highlighted that Tukey's method inherently corrects for multiple comparisons by adjusting the confidence level for each test, thereby limiting the FWER to 5% (*α* = 0.05) [16].

## 3 Results

### 3.1 Analysis of Chemical Components of PNID

**Figure 1:**
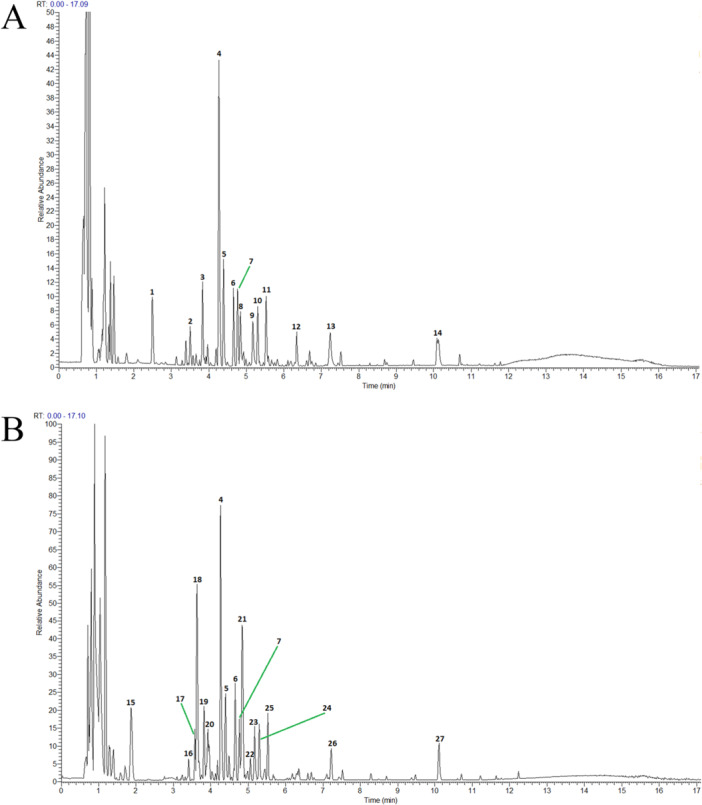
Identification of PNID compounds. (A) Base peak chromatogram of PNID in positive ion mode. (B) Base peak chromatogram of PNID in negative ion mode.

The primary chemical constituents of PNID were characterized using UHPLC‐HRMS. Figure 1 showed the base peak chromatograms acquired in both positive and negative ion modes, facilitating the identification of prominent chromatographic peaks. The chemical profile of PNID was predominantly comprised of organic oxygen compounds, flavonoids, indole and its derivatives, isopentenols, carboxylic acids, isoflavones, and lipids. Notably, PNID contained a diverse array of anti‐inflammatory compounds, including puerarin, albiflorin, and liquiritigenin. Chemical information on these constituents was provided in Table S1.

### 3.2 PNID Alleviated Motor Function in PD Mice

**Figure 2:**
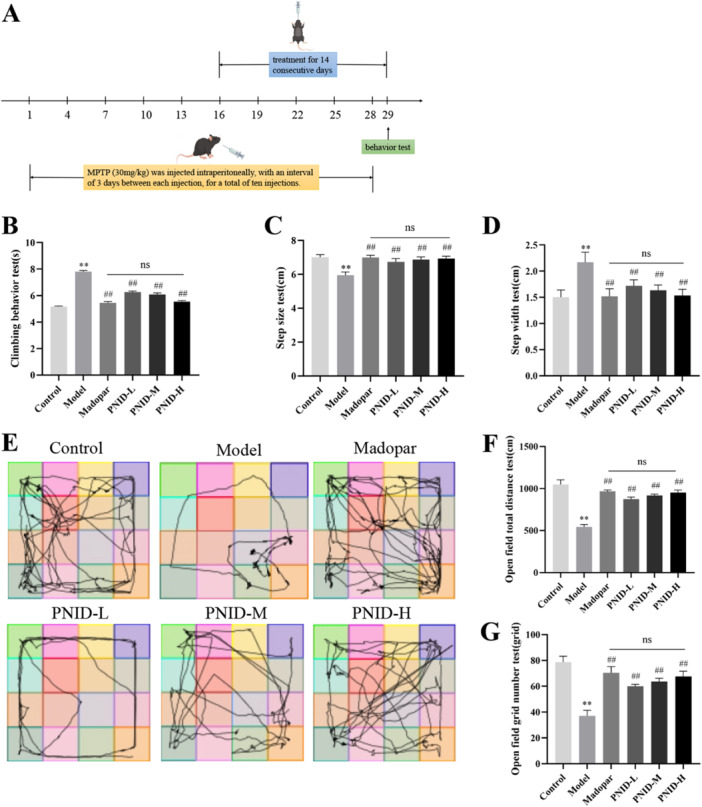
Behavioral assessments revealed that PNID ameliorated motor impairments induced by MPTP in PD mice. Animals were divided into six distinct groups: a control group, a model group, a Madopar group, and three PNID‐administered groups (designated as PNID‐L, PNID‐M, and PNID‐H for low, medium, and high doses, respectively). (A) Experimental design schematic diagram. (B) Pole test. (C) Step size test. (D) Step width test. (E) Open field trajectory. (F) Open field total distance test. (G) Open field grid number test. Data were shown as the mean ± SD. Compared with the control group, **p < 0.01; compared with the model group, ^##^ *p* < 0.01; Statistical analysis was conducted through analysis of one‐way ANOVA and Tukey's multiple comparisons test; *n* = 8 for each group.

Behavioral tests, such as pole climbing, the open‐field experiment, and gait analysis, were conducted to detect motor coordination in mice. Mice in the model group showed motor dysfunction, including a longer climbing time, a longer step width, a shorter step length, a shorter open field crawling distance, and a lower number of grids crossed (*p* < 0.01) compared with the control group. However, PNID alleviated motor impairment in a dose‐dependent manner, especially in the PNID‐H group (*p* < 0.01) compared with the model group, presenting similar behavior to the Madopar group (*p* > 0.05) (Figure 2B–G). These results showed the potential therapeutic benefits of PNID in mitigating motor coordination deficits in the mouse model.

### 3.3 PNID Alleviated Neuron Damage in PD Mice

**Figure 3:**
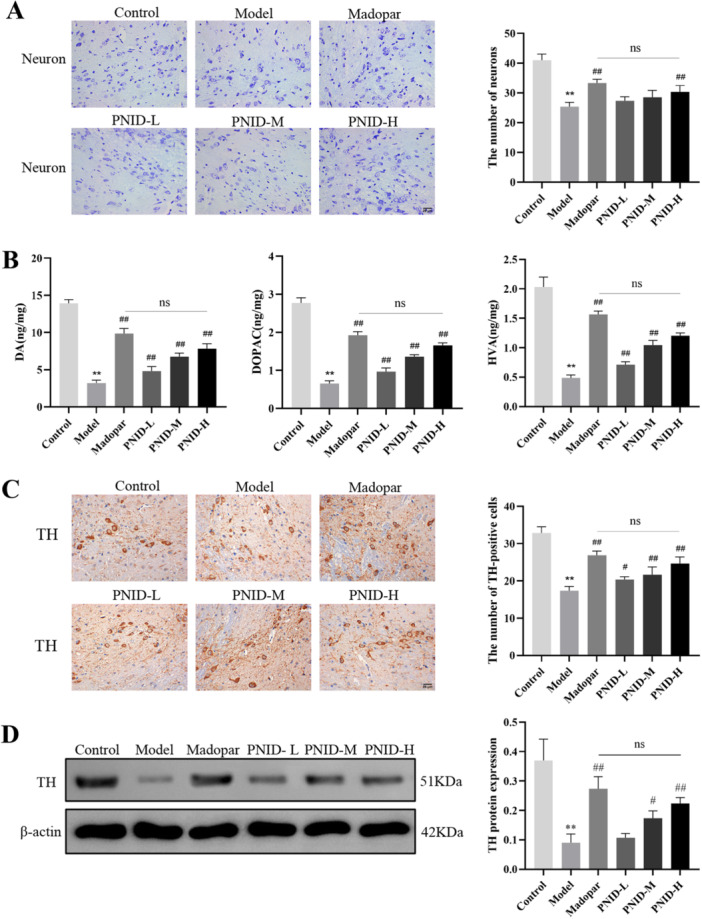
PNID exhibited neuroprotective effects in PD mice by mitigating neurological damage. (A) Neuron Nissl staining (×400 magnification, scale bar: 25 μm). (B) DA, DOPAC, and HVA were conducted using HPLC techniques. (C) Immunohistochemical staining was detected to evaluate the expression of TH (×400 magnification, scale bar: 25 μm). (D) Western blot was used to evaluate the expression of TH. Data were shown as the mean ± SD. Compared with the control group, **p < 0.01; compared with the model group, ^#^ *p* < 0.05 and ^##^ *p* < 0.01; Statistical analysis was conducted through analysis of one‐way ANOVA and Tukey's multiple comparisons test; *n* = 6 for each group.

Neuron damage and loss are core pathological features of PD [1]. Nissl staining was used to evaluate the expression of neurons in midbrain SNpc. Compared with the control group, the expression of neurons in the model group was markedly declined (*p* < 0.01). PNID reduced neuron damage in a dose‐dependent manner (Figure 3A). The reduction of neurons in the PNID‐H group was similar to that in the Madopar group (*p* > 0.05).

### 3.4 PNID Enhanced the Levels of DA and Its Metabolites in PD Mice

DA, acting as a neurotransmitter, plays a crucial role in regulating motor equilibrium in individuals with PD [17]. HPLC was utilized to quantify the striatal concentrations of DA and its metabolites, DOPAC and HVA. Results showed a substantial reduction in the levels of DA, DOPAC, and HVA in the model group relative to the control group (*p* < 0.01). In contrast, the PNID‐H group exhibited significantly elevated levels of DA, DOPAC, and HVA compared to the model group (*p* < 0.01). Notably, the concentrations of these biomarkers in the PNID‐H group were indistinguishable from those in the Madopar group, indicating comparable therapeutic efficacy (*p* > 0.05) (Figure 3B).

### 3.5 PNID Enhanced Expression of TH in PD Mice

TH is an important marker of dopaminergic neurons [18]. The expression of TH in SNpc was detected by immunohistochemistry and western blot. TH expression in the model group was lower (*p* < 0.01) than in the control group. The decrease in TH expression was suppressed in the PNID‐H group (*p* < 0.01). The expression of TH was similar in the PNID‐H and Madopar groups (*p* > 0.05) (Figure 3C,D).

### 3.6 PNID Reduced BBB Damage

**Figure 4:**
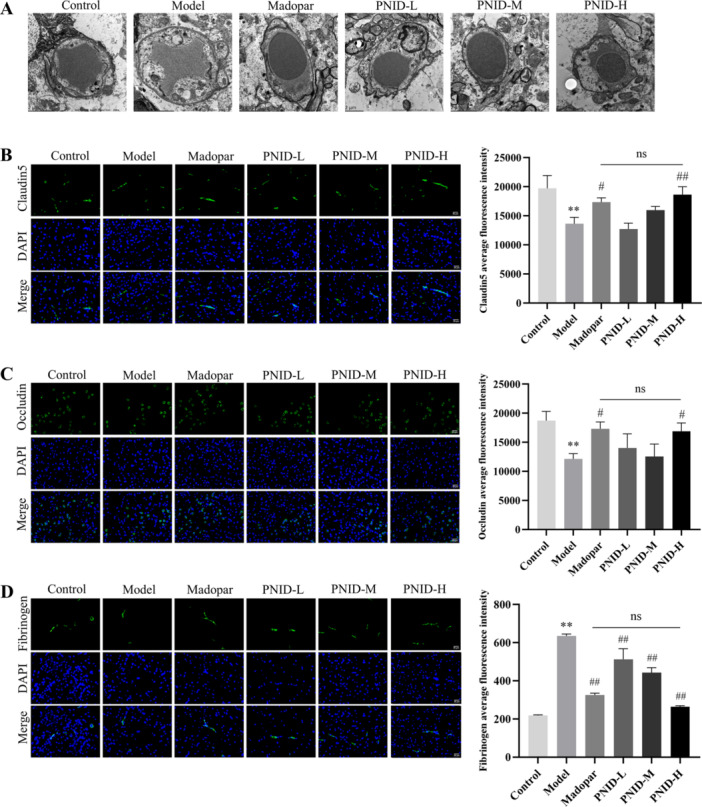
PNID exhibited protective effects on the BBB integrity and reduced fibrinogen accumulation in PD mice. (A) Ultrastructural examination of the BBB in PD mice was observed by using transmission electron microscopy (magnification: 10k×; scale bar: 2 μm). (B) The endothelial tight junction marker claudin‐5 was evaluated through immunofluorescence staining (×400 magnification, scale bar: 25 μm). (C) The expression of endothelial tight junction marker occludin was assessed by immunofluorescence staining (×400 magnification, scale bar: 25 μm). (D) Fibrinogen was detected by immunofluorescence staining (×400 magnification, scale bar: 25 μm). Data were shown as the mean ± SD. Compared with the control group, **p < 0.01; compared with the model group, ^#^ *p* < 0.05 and ^##^ *p* < 0.01; Statistical analysis was conducted through analysis of one‐way ANOVA and Tukey's multiple comparisons test; *n* = 6 for each group.

BBB disruption represents a pivotal pathological mechanism underlying PD [19]. Upon the impairment of the BBB, endothelial cellular integrity and permeability are compromised, accompanied by a downregulation in the expression of tight junction proteins, specifically occludin and claudin‐5 [20, 21]. To quantify the extent of BBB disruption, transmission electron microscopy was employed to scrutinize the features of the BBB, while immunofluorescence techniques were utilized to detect claudin‐5 and occludin. In the model group, notable disruptions were observed, with loose or disrupted tight junctions within the BBB and a thin, discontinuous basement membrane. Conversely, in the PNID‐H group, the BBB exhibited tight and continuous junctions, accompanied by an intact basement membrane (Figure 4A). When compared to the control group, a statistically significant decrease in the expression of both claudin‐5 and occludin was evident in the model group (*p* < 0.01). However, in the PNID‐H group, a significant upregulation in the expression of these tight junction proteins was observed relative to the model group (*p* < 0.05). Notably, the expression of claudin‐5 and occludin were comparable between the PNID‐H and Madopar groups, indicating no significant difference (*p* > 0.05) (Figure 4B,C).

### 3.7 PNID Decreased Fibrinogen Deposition

Fibrinogen accumulates within brain tissue when the BBB undergoes disruption [22]. This accumulated fibrinogen selectively stimulates microglia, leading to the induction of neuroinflammatory responses [23]. To assess fibrinogen expression specifically within the SNpc of the midbrain, immunofluorescence techniques were employed. Our findings revealed a statistically significant elevation in fibrinogen deposition within the model group compared to the control group (*p* < 0.01). Notably, treatment with PNID exhibited a dose‐responsive reduction in fibrinogen deposition, with the most prominent effect observed in the PNID‐H group (*p* < 0.01). Furthermore, the degree of fibrinogen deposition in the PNID‐H group was indistinguishable from that of the Madopar group, as evidenced by a nonsignificant difference between the two groups (*p* > 0.05) (Figure 4D).

### 3.8 PNID Inhibited Microglial Activation and Modulated Polarization

**Figure 5:**
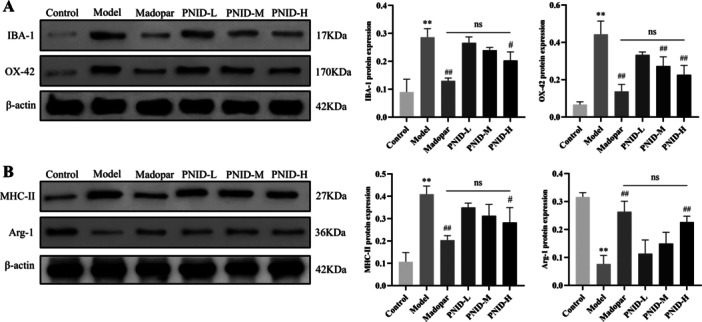
PNID exerted suppressive effects on microglial activation and modulated their polarization states. (A) Western blot quantifies the expression of IBA‐1 and OX42 proteins. (B) Western blot quantifies the expression of MHC‐II and Arg‐1 proteins. Data were shown as the mean ± SD. Compared with the control group, **p < 0.01; compared with the model group, ^#^ *p* < 0.05 and ^##^ *p* < 0.01; Statistical analysis was conducted through analysis of one‐way ANOVA and Tukey's multiple comparisons test; *n* = 6 for each group.

Microglia play a crucial part in the neuroinflammatory response in PD [24], and IBA‐1 and OX42 are specific markers of microglia [25, 26]. To quantify the activation state of microglia, we conducted a western blot analysis to assess the protein of IBA‐1 and OX42 within the SNpc of the midbrain. Results showed robust upregulation of both IBA‐1 and OX42 protein expression in the model group, relative to the control group (*p* < 0.01). Furthermore, a statistically significant difference was noted in the expression levels of these microglial markers between the PNID‐H group and the model group (*p* < 0.05). As expected, the expression of IBA‐1 and OX42 in the PNID‐H group was comparable to those observed in the Madopar group (*p* > 0.05) (Figure 5A).

Activated microglia exhibit distinct M1 and M2 phenotypes, characterized by unique molecular signatures [27]. Specifically, MHC‐II serves as a hallmark of the M1 phenotype, whereas Arg‐1 is indicative of the M2 phenotype [28]. To investigate the impact of these microglial phenotypes, we employed western blot analysis to quantify the expression of MHC‐II and Arg‐1. Our findings revealed that, in comparison to the control group, the model group showed a significant elevation in MHC‐II protein expression and a concurrent reduction in Arg‐1 protein expression (*p* < 0.01). Notably, PNID treatment was found to attenuate MHC‐II expression and augment Arg‐1 expression, with remarkable effects observed in the PNID‐H group (*p* < 0.05). Furthermore, the expression of both MHC‐II and Arg‐1 was comparable between the PNID‐H and Madopar groups (*p *> 0.05) (Figure 5B).

Collectively, these results showed that PNID exerted inhibitory effects on microglial activation and modulated the polarization state of microglia, shifting the balance toward a more anti‐inflammatory M2 phenotype.

### 3.9 PNID Regulated Inflammatory Factors

**Figure 6:**
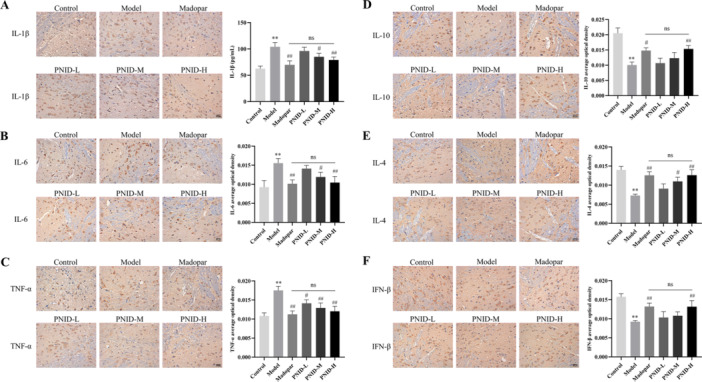
PNID regulated the expression of different types of inflammatory factors. (A) Immunohistochemical staining was utilized to detect the expression of IL‐1β in the midbrain of mice (×400 magnification, scale bar: 25 μm). (B) Immunohistochemical staining was utilized to detect the expression of IL‐6 in the midbrain of mice (×400 magnification, scale bar: 25 μm). (C) Immunohistochemical staining was utilized to detect the expression of TNF‐α in the midbrain of mice (×400 magnification, scale bar: 25 μm). (D) Immunohistochemical staining was utilized to detect the expression of IL‐10 in the midbrain of mice (×400 magnification, scale bar: 25 μm). (E) Immunohistochemical staining was utilized to detect the expression of IL‐4 in the midbrain of mice (×400 magnification, scale bar: 25 μm). (F) Immunohistochemical staining was utilized to detect the expression of IFN‐β in the midbrain of mice (×400 magnification, scale bar: 25 μm). Data were shown as the mean ± SD. Compared with the control group, **p < 0.01; compared with the model group, ^#^ *p* < 0.05 and ^##^ *p* < 0.01; Statistical analysis was conducted through analysis of one‐way ANOVA and Tukey's multiple comparisons test; *n* = 6 for each group.

Activated microglia of the M1 phenotype secrete a suite of pro‐inflammatory factors, whereas activated M2 microglia produce anti‐inflammatory factors [29]. To gain deeper insights into the inflammatory response profile, immunohistochemical staining techniques were utilized to assess the expression of inflammatory cytokines within the SNpc. The results indicated a robust upregulation of pro‐inflammatory cytokines, including IL‐1β, IL‐6, and TNF‐α, in the model group compared to the control group (*p* < 0.01). Conversely, the expression levels of anti‐inflammatory cytokines such as IL‐10, IL‐4, and IFN‐β were significantly downregulated in the model group (*p* < 0.01). Notably, within the PNID‐H group, there was a significant augmentation of pro‐inflammatory cytokine levels accompanied by a reduction in anti‐inflammatory cytokine levels (*p* < 0.01). However, when comparing the PNID‐H group with the Madopar group, no statistically significant differences in the levels of these inflammatory cytokines were observed (*p* > 0.05) (Figure 6A–F). These findings suggested that PNID treatment modulated the inflammatory milieu in the SNpc, particularly in the PNID‐H group, with effects comparable to those of Madopar.

## 4 Discussion

**Figure 7:**
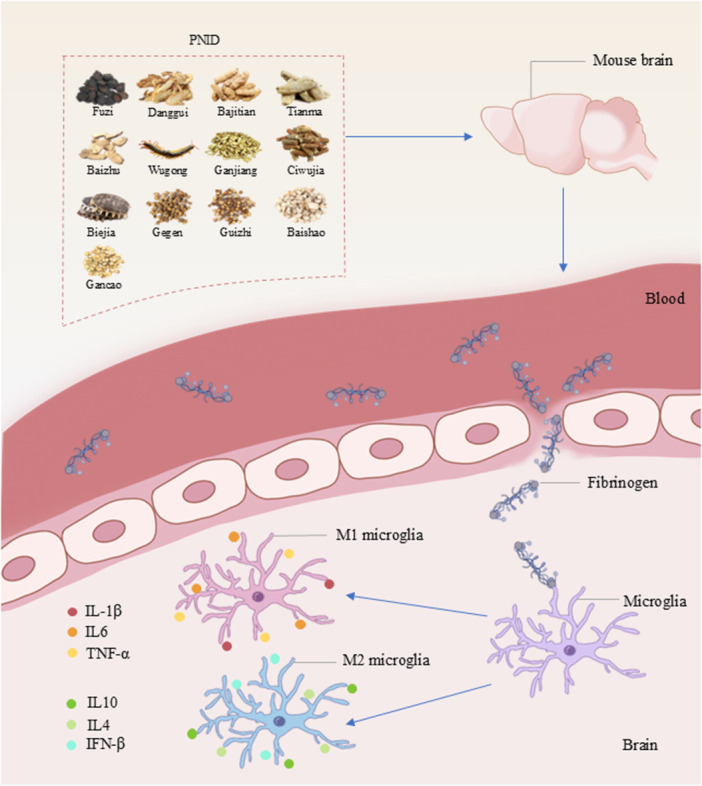
Diagram of the mechanism of PNID in PD.

We assessed the efficacy of PNID in PD mice and explored its possible mechanism in neuroinflammation. PNID significantly alleviated limb tremors and body curling and enhanced locomotor balance in PD mice. Besides, PNID treatment significantly alleviated damage to neurons and the BBB, decreased fibrinogen deposition, inhibited microglial activation, reduced the expression of pro‐inflammatory cytokines (IL‐1β, IL‐6, and TNF‐α), and enhanced the expression of pro‐inflammatory cytokines (IL‐4, IL‐10, and IFN‐β). This study suggested that PNID may be an effective complementary and alternative drug for the treatment of PD. PNID may alleviate microglial activation‐mediated neuroinflammation by inhibiting fibrinogen deposition, thereby inhibiting pathological progression and reducing motor dysfunction (Figure 7).

Neuron loss and reduced striatum DA content are core pathological features of PD [1]. TH, as the key rate‐limiting enzyme of DA, is an important marker of DA neurons, which can evaluate the degree of dopaminergic neuron damage and the efficacy of treatment [18]. In this study, the expression of neurons, TH, and DA declined in PD model mice. After PNID intervention, mice showed a marked improvement in motor function, a decline in neuron damage, and increased TH, DA, DOPAC, and HVA expression, which indicated that the therapeutic efficacy of PNID in PD was significant.

The BBB is a key barrier that blocks harmful substances from entering the brain, which plays an essential part in maintaining the stability of the nervous system [30]. Occludin and claudin‐5 are marker proteins of endothelial cell tight junctions in the BBB [31]. Fibrinogen can enter the CNS through the BBB in large quantities when the BBB is damaged; subsequently, fibrinogen is deposited in the brain through blood vessels, accelerating damage to vessels and neurons [22]. Fibrinogen is a marker of the neuroinflammatory response and a therapeutic target for treating inflammation [32] that specifically combines with and activates the CD11b/CD18 integrin receptor of the microglia, thereby promoting an inflammatory response [33]. This study showed that the expression of occludin and claudin‐5 was increased and fibrinogen deposition was decreased after PNID treatment. PNID may exert a protective effect on the BBB by reducing the damage to the tight junctions of endothelial cells, thereby inhibiting fibrinogen deposition in brain tissue.

Neuroinflammation is one of the important pathogenic mechanisms of PD [34, 35, 36]. A recent study has demonstrated the intense activation of glial cells and the apoptotic death phenomenon accompanied by the degeneration of dopaminergic neurons, further emphasizing the crucial role of neuroinflammation in the pathological process of PD [3]. Microglia are major brain cells that are involved in neuroinflammatory responses and participate in pro‐inflammatory and anti‐inflammatory signal transduction [27]. Over‐activated microglia have an abundance of pro‐inflammatory factors, which can destroy dopaminergic neurons [37] and the BBB [38]. The different phenotypes of activated microglia are classified as M1 or M2. M1 microglia induce inflammation, release pro‐inflammatory factors such as IL‐1β and IL‐6, and damage neurons [39]. M2 microglia exert anti‐inflammatory effects and release anti‐inflammatory factors such as IL‐1β and IL‐4 to alleviate the inflammatory response [40]. In this study, we discovered that PNID restrained microglial activation while regulating the phenotype from the M1 to the M2, reducing the protein expression of OX42, IBA‐1, and MHC‐II and increasing the protein expression of Arg‐1. PNID enhanced the expression of anti‐inflammatory factors (IL‐4, IL‐1β, and IL‐10) and decreased the expression of pro‐inflammatory factors (IL‐6, IL‐1β, and TNF‐α). Inhibition of microglial activation and regulation of inflammatory factors may be the mechanisms by which PNID reduces the inflammatory response.

The result of the research indicated that PNID reduced fibrinogen deposition and decreased microglia‐mediated neuroinflammation, suggesting a neuroprotective effect of PNID. These findings highlight the potential clinical implications of PNID in treating neuroinflammatory and PD. Future studies should focus on validating these effects in preclinical models with long‐term outcome assessments, exploring their combinatory potential with existing neuroprotective agents. A recent study demonstrated that, contrary to the traditional view of PD pathology being centered on neuronal cell bodies, axons represent earlier and more critical sites of lesion [41]. Although our study primarily focused on neuroinflammation and neuronal loss, future investigations using this model should include detailed evaluations of axonal lesions within the substantia nigra–striatum pathway, for example, by analyzing axonal morphology, synaptic protein levels, and transport proteins such as DAT and VMAT2, to contribute to a deeper understanding of PD mechanisms.

## 5 Conclusions

This study suggested that PNID has potential as an alternative therapeutic option in the management of PD. Specifically, PNID may exert its beneficial effects by mitigating microglial activation‐induced neuroinflammation, accomplished through the inhibition of fibrinogen accumulation, thereby interrupting the progression of neuropathological processes in PD mice.

## Author Contributions

**Yan‐Jun Chen:** conceptualization, data curation, formal analysis, investigation, methodology, software, visualization, writing – original draft. **Jing‐Wen Chen:** formal analysis, investigation, data curation. **Ming‐Rong Xie:** software, visualization. **Rui‐Zhen Wang:** formal analysis, investigation. **Yu‐Ling Wan:** investigation. **Jie Zeng:** investigation. **Bing‐Wu Zhong:** conceptualization, supervision, writing – review and editing. **Sheng‐Qiang Zhou:** conceptualization, supervision, validation, writing – review and editing. **Fang Liu:** conceptualization, resources, validation, writing – review and editing, supervision.

## Ethics Statement

The Experimental Animal Ethics Committee of Hunan University of Chinese Medicine has approved the conduct of this experiment (Ethical Approval Number: LL2022060101).

## Consent

All authors have provided their consent for publication of the manuscript.

## Conflicts of Interest

The authors declare no conflicts of interest.

## Supporting information

Supplementary Table 1: The main chemical components of PNID.

## Data Availability

The data used to support the findings of this study are available from the corresponding author upon request.
