# Supplementary material for: Reducing Neuroinflammation via Inhibition of Fibrinogen Deposition and Microglial Activation as an Underlying Mechanism of Paning I Decoction in Ameliorating Parkinson's Disease Symptoms
Source: Immun Inflamm Dis. 2026 Feb 5;14(2):e70310. doi: 10.1002/iid3.70310 (PMC12877312; doi:10.1002/iid3.70310)
Supplement: Supplementary file 1 — Supplementary Table 1: The main chemical components of PNID. [file IID3-14-e70310-s001.docx]

**Supplementary Table 1: The main chemical components of PNID.**

| **Chemical name** | **Formula** | **RT (min)** | **Mulecular Weight (Da)** |
| --- | --- | --- | --- |
| Phenylalanine | C_9_H_11_NO_2_ | 2.48 | 165.19 |
| 3-Indolyllacticacid | C_11_H_11_NO_3_ | 3.51 | 205.21 |
| Vitexin | C_21_H_20_O_10_ | 3.84 | 432.4 |
| Puerarin | C_21_H_20_O_9_ | 4.28 | 416.4 |
| Puerariaglycoside3 | C_22_H_22_O_10_ | 4.41 | 446.4 |
| Albiflorin | C_23_H_28_O_11_ | 4.67 | 480.5 |
| Daidzin | C_21_H_20_O_9_ | 4.78 | 416.4 |
| .gamma.-CEHC | C_15_H_20_O_4_ | 4.85 | 254.32 |
| Isoliquiritigen | C_15_H_12_O_4_ | 5.18 | 256.25 |
| Liquiritigenin | C_15_H_12_O_4_ | 5.31 | 256.25 |
| 6''-O-Malonyldaidzin | C_24_H_22_O_12_ | 5.52 | 502.4 |
| Benzoylmesaconine | C_31_H_43_NO_10_ | 6.35 | 589.7 |
| Daidzein | C_15_H_10_O_4_ | 7.23 | 254.24 |
| 18.beta.-Glycyrrhetinicacid | C_30_H_46_O_4_ | 10.1 | 470.7 |
| 3-Galloylgallocatechin | C_22_H_18_O_11_ | 1.92 | 458.4 |
| Violanthin | C_27_H_30_O_14_ | 3.38 | 478.5 |
| Apigenin-8-c-glucoside | C_21_H_20_O_10_ | 3.81 | 432.4 |
| Benzenepropanoicacid,4-hydroxy- | C_9_H_10_O_3_ | 3.91 | 166.17 |
| 3-Hydroxysubericacid | C_8_H_14_O_5_ | 5.06 | 190.19 |
| Liguiritigenin-7-O-beta-D-apiosyl-4'-O-beta-D-glucoside | C_26_H_30_O_13_ | 5.17 | 550.5 |
| Liquiritin | C_21_H_22_O_9_ | 5.3 | 418.4 |
| 7,8-Dihydroxyflavone | C_15_H_10_O_4_ | 5.52 | 254.24 |
| Apigeninidin | C_15_H_11_ClO_4_ | 7.22 | 290.7 |
| Glycyrrhizinatedipotassium | C_42_H_60_K_2_O_16_ | 10.12 | 899.1 |
| Parishine | C_19_H_24_O_13_ | 3.58 | 460.4 |
| Paeoniflorin | C_23_H_28_O_11_ | 4.85 | 480.5 |
